# The level of agreement regarding patient disposition between emergency physicians and consultants in the emergency department

**DOI:** 10.1186/1865-1380-6-22

**Published:** 2013-07-08

**Authors:** Mitsunaga Iwata, Katsuo Yamanaka, Yoshimi Kitagawa

**Affiliations:** 1 Department of Emergency and General Internal Medicine, Fujita Health University, 1-98 Dengakugakubo Kutsukake-cho, Toyoake Aichi, 470-1192, Japan; 2 Department of Emergency Medicine, Nagoya Ekisaikai Hospital, 4-66 Shonenn-cho Nakagawa-ku, Nagoya Aichi, 468-0042, Japan

**Keywords:** Consultation, Emergency physician, Consultant, Disposition

## Abstract

**Background:**

Consultation is a common and important aspect of emergency medicine practice. We examined the frequency of consultations, the level of agreement and factors of disagreement with regard to the disposition of patients who visited two emergency departments (EDs) of tertiary care hospitals in Japan.

**Findings:**

We analyzed 3,503 consecutive patients who visited two EDs in Japan during a 3-month period. The initial diagnosis in the ED, the presence of consultation, and the patient disposition following the ED visit were recorded. At least one consultation was requested in 34.7% of the patients (1,215/3,503), and 88% of these patients were admitted to the hospital (1,063/1,215). Consultants and emergency physicians (EPs) agreed on patient disposition 95% of the time (1,153/1,215), with κ = 0.76 (95% CI 0.70-0.82). Opinions about disposition were discordant in 5% of the patients consulted upon. In 63% of those cases, patients were not diagnosed in the ED.

**Conclusion:**

Consultants and EPs agreed on patient disposition in most cases. In more than half of the cases in which disagreements arose between EPs and consultants, the EPs were not able to reach an initial diagnosis. Further studies are needed to examine the association between disagreements in disposition and adverse outcomes.

## Findings

### Background

Consultation in the emergency department (ED) is the process by which emergency physicians (EPs) request other specialists (consultants) to participate in the care of an ED patient. In order to ensure the safe ongoing care of ED patients, appropriate communication is essential.

Though consultation is a common and important aspect of emergency medicine practice, little research has been conducted on this topic in emergency medicine [1].

Few studies have examined the consultation outcomes for special populations of ED presentations [2-4]. Moreover, the level of agreement between consultants and EPs regarding final patient dispositions was examined in only one study in Canada [5], and the factors underlying any disagreements were unknown.

We examined the frequency of consultations, the level of agreement, and the factors of disagreement with regard to disposition in patients who visited two EDs of tertiary care hospitals in Japan.

### Methods

This study was conducted on consecutive patients who visited EDs of Fujita Health University Hospital (FHUH) and Nagoya Ekisaikai Hospital (NEH) during the daytime (8:30 a.m.-17:00 p.m.) between 1 October and 28 December 2012. Both hospitals are regional referral centers for a variety of emergency medical services and can provide almost every type of specialist care. In our study, EPs and consultants were defined board-certified specialists and post-graduate-year (PGY) 3 and 4 residents. PGY 1 and 2 residents must consult with EPs in all cases, and they can’t consult with consultants directly. In both EDs, consultation was requested when EPs judged that a patient should be admitted or should be followed closely by specialists in outpatient settings during the day. We excluded patients who visited EDs at night (17:00 p.m.-8:30 a.m.) because EPs could admit them without consultation and consultation was performed the next day. During the study interval, a total of 15,850 patients visited EDs (FHUH: 6,416, NEH: 9,434), but 12,332 patients who visited EDs at night and 15 patients whose consultation information was missing in the electronic medical recoding system were excluded. The final study population included 3,503 patients.

From each ED record (electronic medical recoding system), we extracted the initial diagnosis in the ED, the presence of consultation, and the patient disposition following the ED visit retrospectively. Concerning the consultation outcomes, one board-certified emergency physician judged “disagreement” when opinions about disposition were discordant between EPs and consultants.

All analyses were performed using the Statistical Package for the Social Sciences (SPSS). The chi-squared test and Mann-Whitney U test were used to compare differences between patients receiving consultations and those without consultations. The κ statistic was used to evaluate the level of agreement among EPs and consultants regarding disposition. A κ score in the range from 0.0 to 0.40 was considered poor, 0.41 to 0.60 moderate, and 0.61 to 0.80 substantial agreement [6].

### Ethical issue

The study was approved by the Fujita Health University Research Ethics Board.

### Results

During the study interval, at least one consultation was requested in 34.7% of the study patients (1215/3503), with a total of 1,318 consultations requested in those 1,215 patients. Six percent of those patients had two consultations, while 1% had more than two consultations requested.

The characteristics of the patients and the factors associated with the consultation are described in Table 1. The consultation proportions were similar between males and females. Consultations occurred more frequently in patients who arrived by ambulance and had cardiovascular problems.

**Table 1:** Characteristics of 3,503 patients in two emergency departments

	**Patients with consultations (*n *= 1,215)**	**Patients without consultations (*n *= 2,288)**	***p *value**	**Frequency of consultations (%)**
**Mean age (SD)**	53.8 (26.4)	49.4 (28.6)	n.s	
**Male sex (%)**	628 (51.7)	1,249 (54.6)	n.s	
**EMS arrival (%)**	1,045 (86.0)	326 (14.2)	*P* < 0.001	
**Initial diagnosis in the ED**				
Respiratory problems	412 (33.9)	691 (30.2)		37.3
Neurological problems	83 (6.8)	128 (5.6)		39.3
Gastrointestinal problems	398 (32.8)	680 (29.7)		36.9
Cardiovascular problems	106 (8.7)	46 (2.0)		69.7
Musculoskeletal problems	22 (1.8)	131 (5.7)		14.3
Psychiatric problems	13 (1.1)	149 (6.5)		8
Other medical problems	24 (2.0)	48 (2.1)		33
Cases in which emergency physicians can’t achieve diagnosis	92 (7.6)	222 (9.7)		29.2
Trauma	65 (5.3)	193 (8.4)		25.1

Consultants and EPs agreed on patient disposition 95% of the time (1,153/1,215), with κ = 0.76 (95% CI 0.70-0.82; Table 2). Eighty-eight percent of the patients consulted upon were admitted to the hospital (1,063/1,215).

**Table 2:** Level of agreement between the emergency physician and consultant regarding patient disposition

		**Emergency physician**	
		**Admission**	**Discharge**
**Consultant**	Admission	1039	24
	Discharge	38	114

The initial diagnosis in 62 cases in which disagreements arose between the EP and consultant are described in Table 3. In 63% of those cases, the patients were not diagnosed in the ED (39/62).

**Table 3:** Initial diagnosis in 62 cases in which disagreements arose between the emergency physician and the consultant

**Cases in which patients were not diagnosed in the ED**	**39**
Fever of unknown origin	18
Weakness of unknown origin	8
Syncope of unknown origin	6
Chest pain of unknown origin	4
Acute low back pain of unknown origin	3
**Infectious disease**	13
Pneumonia	6
Urinary tract infection	5
Cellulitis	2
**Respiratory problems**	3
**Trauma**	7

### Discussion

To our knowledge, this is the first study that has reported the frequency of consultation and, the level of agreement and factors of disagreement with regard to patient disposition in Japanese EDs.

EPs requested consultation in about one-third of ED patients, with most of the patients undergoing consultation being admitted. The frequency of consultations was similar to those of previous studies. However, the proportion of those patients admitted was much higher than that shown in a previous study in which the proportion was 50%. This may be a result of EPs in Japan consulting with another physician when they judged that a patient should be admitted.

Though a previous study has reported that when compared with physicians from inpatient services (i.e., family medicine or internal medicine), EPs would admit more and discharge fewer patients from the ED [7], the level of agreement among EPs and consultants regarding disposition was substantial in our study and similar to the results of a previous study in Canadian EDs [5]. This is a very important finding, since EPs are required to obtain the consent of consultants to admit a patient to the hospital in most cases in Japan.

It was thought that disagreements sometimes arise between EPs and consultants as to the patients’ most appropriate disposition, but no study has examined the differences in patient disposition. In our study, opinions about disposition were discordant for 5% of patients undergoing consultation. In 63% of those cases, the EPs were not able to reach an initial diagnosis in the ED. This is likely because patients with clear-cut illnesses are usually not problematic, but, in other cases, the decision to admit may be made on secondary, less tangible criteria, including the assessment of risk for adverse outcomes, whether the patient has accessible physician follow-up, whether the patient has the means to return to the ED, how much competent support the patient has at home, and whether there are sufficient health care options available in the community. Further studies are needed to assess the rates of ED return for patients who were discharged and for whom the EP and consultant opinions were discordant. Additionally, in 39% of those cases, EPs determined that patient discharge with close follow-up was possible, whereas consultants recommended admission. Of these cases, there remains some possibility that should these patients be discharged without consultation, they would have ended up returning to the hospital unexpectedly or having delay in treatment. Therefore, EPs should follow up on the patients even after they have been admitted.

The limitations of this study are as follows, and a well-designed multi-institutional observation study is needed to address this issue further.

1. This study was a retrospective study, and data were missing in 15 cases.

2. There was only one person determining disagreement in disposition.

3. Sampling restricted to daytime hours because of local procedures and lack of MD form completion were major limitations.

4. The variability of consultation based on the condition [high: respiratory (34%); low: psychiatric (1%)] was likely to depend on the ED characteristics (community vs. tertiary care), and our findings may not be generalizable to other EDs across the nation.

5. Disagreements in disposition might be associated with adverse outcomes (e.g., prolonged length of stay in the ED [8] or delay in treatment), but we couldn’t provide times parameters for the ED journey in this study.

6. This study was performed at two institutions, and our findings may not be generalizable to other EDs across the nation.

### Conclusions

This is the first study reporting on consultation in the Japanese ED setting. Consultants and EPs agreed on patient disposition in most cases. In more than half of the cases in which disagreements arose between EPs and consultants, the EPs were not able to reach an initial diagnosis. Further studies are needed to examine the association between disagreements in disposition and adverse outcomes.

## Abbreviations

ED: emergency department; EP: emergency physician; FHUH: Fujita Health University Hospital; NEH: Nagoya Ekisaikai Hospital; PGY: post-graduate-year.

## Competing interests

The authors declare that they have no competing financial or non-financial interests.

## Authors’ contributions

Mitsunaga Iwata conceived of the study, participated in its design and coordination, and drafted the manuscript. Katsuo Yamanaka participated in its design and revised the manuscript. Yoshimi Kitagawa participated in its design. All authors read and approved the final manuscript.
